# Prevalence and Severity of Anaemia Stratified by Age and Gender in Rural India

**DOI:** 10.1155/2014/176182

**Published:** 2014-12-04

**Authors:** Gerardo Alvarez-Uria, Praveen K. Naik, Manoranjan Midde, Pradeep S. Yalla, Raghavakalyan Pakam

**Affiliations:** Department of Infectious Diseases, Bathalapalli Rural Development Trust Hospital, Kadiri Road, Bathalapalli, Anantapur District, Andhra Pradesh 515661, India

## Abstract

Anaemia is a major public health problem in India. Although nearly three quarters of the Indian population live in rural areas, the epidemiology of anaemia in rural settings is not well known. We performed a retrospective observational study using routine clinical data from patients attending the out-patient clinics of a rural hospital in India from June 2011 to August 2014. The study included 73,795 determinations of haemoglobin. 49.5% of patients were female. The median haemoglobin concentration was 11.3 g/dL (interquartile range (IQR), 9.8–12.4) in females and 12.5 g/dL (IQR, 10.6–14.2) in males. Anaemia was present in the majority of children <10 years, women after puberty, and older adults. Children <5 years had the highest prevalence of anaemia, especially children aged 1-2 years. The high proportion of microcytic anaemia and the fact that gender differences were only seen after the menarche period in women suggest that iron deficiency was the main cause of anaemia. However, the prevalence of normocytic anaemia increased with age. The results of this study can be used by public health programmes to design target interventions aimed at reducing the huge burden of anaemia in India. Further studies are needed to clarify the aetiology of anaemia among older adults.

## 1. Introduction

According to the World Health Organization (WHO), there are two billion people with anaemia in the world and half of the anaemia is due to iron deficiency [1]. Anaemia is a late indicator of iron deficiency, so it is estimated that the prevalence of iron deficiency is 2.5 times that of anaemia [1, 2]. The estimated prevalence of anaemia in developing countries is 39% in children <5 years, 48% in children 5–14 years, 42% in women 15–59 years, 30% in men 15–59 years, and 45% in adults >60 years [1]. These staggering figures have important economic and health consequences for low- and middle-income countries. Anaemia and iron deficiency lead to substantial physical productivity losses in adults [2]. Iron deficiency during pregnancy is associated with maternal mortality, preterm labour, low birth-weight, and infant mortality [2]. In children, iron deficiency affects cognitive and motor development and increases susceptibility to infections [3].

Anaemia is a major health problem in India. In the 2005-2006 National Family Health Survey (NFHS-3), a household survey aimed at having national and state representative data on population health and nutrition; the prevalence of anaemia was 70% in children aged 6–59 months, 55% in females aged 15–49 years, and 24% in males aged 15–49 years [4]. Although the NFHS-3 showed that the prevalence of anaemia was higher in rural areas, there is a paucity of data about the epidemiology of anaemia in rural settings [5]. The aim of this study is to describe the prevalence of anaemia among patients who attended the outpatient clinics of a rural hospital in Andhra Pradesh, India.

## 2. Methods

### 2.1. Setting

The study was performed in Anantapur, a district situated in the South border of Andhra Pradesh, India. Anantapur has a population of approximately four million people. In Anantapur, 72% of the population live in rural areas and 36% are illiterate [5]. Rural Development Trust General Hospital is a nonprofit 220-bed hospital in Bathalapalli, a rural village in Anantapur. The hospital belongs to a nongovernmental organization called Rural Development Trust.

### 2.2. Study Design

We collected epidemiological data (age and sex) and laboratory data from the hospital database of patients who attended outpatient clinics from June 1, 2011, to August 31, 2014. HIV infected patients were excluded. In patients who had more than one determination of haemoglobin during the study period, only one determination per year of age was allowed in order to avoid repeated measurements in the same patient. We used definitions of anaemia according to recommendation from the WHO (Table 1) [6]. Microcytic anaemia was defined according to cut-offs proposed by the US Centers for Disease Control and Prevention (CDC) (1-2 years: <77 fL; 3–5 years: <79 fL; 6–11 years: <80 fL; 12–15 years: <82 fL; >15 years: <85 fL) [7].

The study was approved by the Hospital Ethical Committee. Statistical analysis was performed using Stata Statistical Software (Stata Corporation. Release 12.1 College Station, Texas, USA).

## 3. Results 

**Figure 1:**
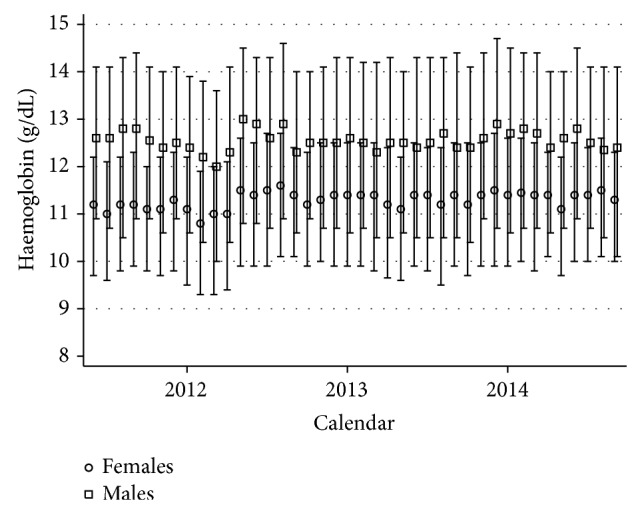
Median and interquartile range of haemoglobin concentration stratified by gender and calendar month.

The study included 73,795 determinations of haemoglobin from 69,440 patients, of which 34,399 (49.45%) were female. The median haemoglobin concentration was 11.8 g/dL (interquartile range (IQR), 10.2–13.3) and the median age was 25 years (IQR, 12–42). The median haemoglobin concentration was 11.3 g/dL (IQR, 9.8–12.4) in females and 12.5 g/dL (IQR, 10.6–14.2) in males. Haemoglobin concentrations did not change significantly during the duration of the study (Figure 1).

**Figure 2:**
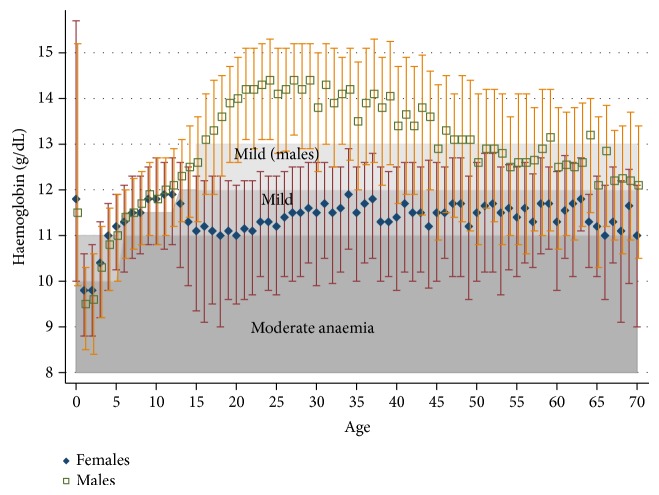
Median and interquartile range of haemoglobin concentration stratified by gender and age.

In Figure 2, we present the median concentration of haemoglobin and interquartile range stratified by age and gender. Children aged 6–30 months had the lowest haemoglobin concentrations. Females and males had similar haemoglobin concentrations until the onset of puberty (around age 13 years). After puberty, females had median concentrations of haemoglobin around 11.5 g/dL, whereas males had a rapid increase in haemoglobin concentrations reaching a plateau of about 14 g/dL at age 20 years and experienced a progressive decline after age 40 years.

**Figure 3:**
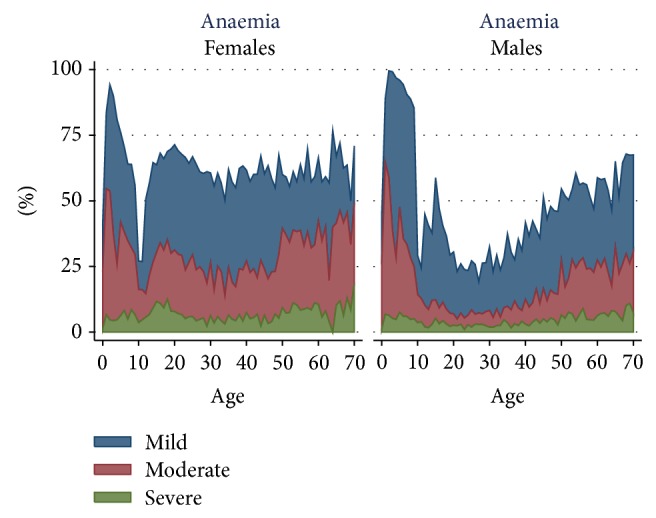
Prevalence of mild, moderate, and severe anaemia by age in males and females.

The prevalence of mild, moderate, and severe anaemia is presented in Figure 3. The highest prevalence of mild and moderate anaemia was seen in children <10 years. The highest prevalence of moderate anaemia was seen in children aged 1-2 years. Both female and male children experienced a rapid improvement in the prepuberty period. After puberty, the prevalence of anaemia was constantly over 50% in females, having older women higher prevalence of moderate and severe anaemia than younger women. Males had a peak of anaemia during puberty and a progressive increase of mild, moderate, and severe anaemia with age.

**Figure 4:**
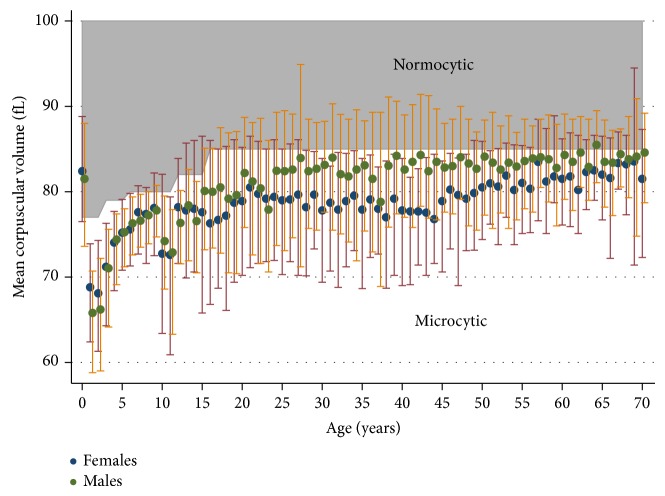
Median and interquartile range of the mean corpuscular volume in patients with anaemia stratified by gender and age.

The median and interquartile range of the mean corpuscular volume (MCV) in patients with anaemia is presented in Figure 4. The vast majority of children with anaemia had low MCV and there were no gender differences. In adults with anaemia, males tended to have a higher MCV than women, but differences reduced with age.

**Figure 5:**
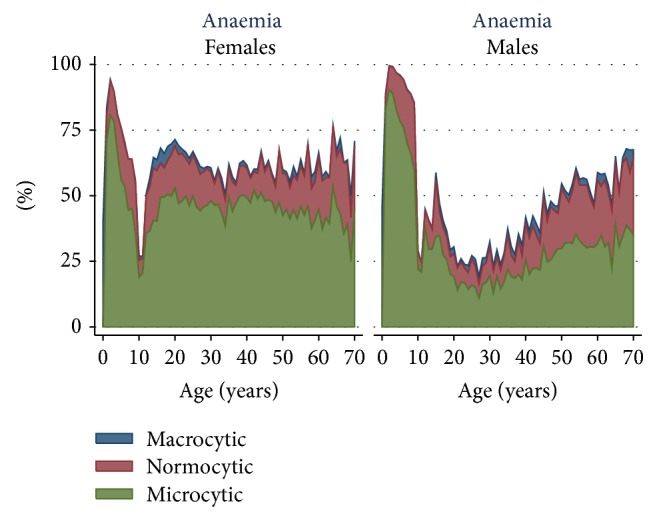
Prevalence of macrocytic, normocytic, and microcytic anaemia by age in males and females.

The prevalence of macrocytic, normocytic, and microcytic anaemia is presented in Figure 5. In general, macrocytic anaemia was rare. While microcytic anaemia was more prevalent in children and women, the proportion of normocytic anaemia increased progressively with age in male adults and women after menopause age.

## 4. Discussion 

In this retrospective observational study using routine clinical data from a large number of patients attending the out-patient clinics of a rural hospital in India, anaemia was present in the majority of children <10 years, women after the onset of puberty, and older adults.

The high proportion of microcytic anaemia and the fact that gender differences were only seen after the menarche period in women indicate that iron deficiency was the main cause of anaemia. In a study of children aged 12–23 months in two rural districts in India, 72% of children with anaemia had low ferritin levels [8]. Other Indian studies have also shown high prevalence of iron deficiency anaemia among young women [9, 10]. The high prevalence of iron deficiency anaemia among women in childbearing age has important public health implications. It is estimated that anaemia accounts for 12.8% of maternal mortality in Asia [11]. Iron requirements are greater in pregnancy, and iron deficiency is associated with maternal death, preterm delivery, and low birth-weight [12, 13]. In India, only 28% of women consume meat, fish, or eggs on a weekly basis [4], and the iron bioavailability of the vegetarian diet is poor [10, 14]. Effective public health programmes aimed at reducing iron deficiency among young women could have a major impact in reducing maternal and infant mortality [15].

The highest prevalence of anaemia was seen in children <10 years, especially in those <5 years. In India, over 95% of children are breastfed [4]. The WHO organization recommends introducing solid and semisolid food at the age of six months because breastfeeding does not suffice to maintain optimal growth after this age. However, at age 6–8 months only 45% of children receiving breastfeeding are given solid or semisolid food [4, 16]. Moreover, only 10% of breastfeeding children and 20% of nonbreastfeeding children aged 6–35 months eat meat, fish, or eggs [4], which are rich in haem iron with high bioavailability [17, 18]. In the NFHS-3, only 14.6% of children aged 6–35 months consumed food rich in iron in the previous 24 hours of the survey [4]. At this age, the effect of iron deficiency on the neurological development can be not totally reversible [3, 19]. Consequently, the Indian Government recommends iron and folic acid supplementations to younger children [20]. However, the programme implementation has been poor due to lack of logistic planning and accountability [20]. In our study, we did not observe an increase in haemoglobin concentrations during the study period suggesting that the programme has not achieved a reduction in the prevalence of anaemia in our setting. Our results are in agreement with other studies in India [21] and indicate that the iron supplementation programme for children aged <24 months should be better monitored.

In this study, we observed an increased prevalence of anaemia with age. Interestingly, the proportion of normocytic anaemia was highest in older adults, suggesting that other causes than iron deficiency might have contributed to the high prevalence of anaemia in this group. Recent studies have shown the poor bioavailability of vitamin B12 in the typical Indian vegetarian diet [14] and substantial prevalence of vitamin B12 deficiency in Indian patients with anaemia [9, 10, 22]. However, new studies investigating the aetiology of anaemia among older adults are needed.

The study has some limitations. We used data from patients coming to the hospital to assess the prevalence of anaemia in the population. This might have led to an overestimation of anaemia in our setting. However, we excluded patients admitted to the hospital, and the prevalence of anaemia was similar to the ones reported in Andhra Pradesh in the NFHS-3 (70.8% in children 6–59 months; 62.9% in females 15–49 years; 23.3% in males 15–49 years) [4].

## 5. Conclusions 

In our rural setting, most patients attending out-patient clinics had anaemia. The highest prevalence of anaemia was seen in children <10 years followed by women and older adults. The vast majority of anaemia cases were microcytic, suggesting that iron deficiency was the main cause of anaemia. However, the prevalence of normocytic anaemia increased with age, so further studies are needed to clarify the cause of anaemia among older adults. The results of this study can be used by public health programmes to design target interventions aimed at reducing the huge burden of anaemia in India.

## Figures and Tables

**Table 1:** Haemoglobin concentrations (g/dL) for the diagnosis of anaemia and assessment of severity according to the World Health Organization.

Age	Mild	Moderate	Severe
6–59 months	10–10.9	7–9.9	<7
5–11 years	11–11.4	8–10.9	<8
12–14 years	11–11.9	8–10.9	<8
Female >14 years	11–11.9	8–10.9	<8
Male >14 years	11–12.9	8–10.9	<8
